# Case Series of Post-Thrombolysis Patients Undergoing Hemicraniectomy for Malignant Anterior Circulation Ischaemic Stroke

**DOI:** 10.1155/2011/254569

**Published:** 2011-04-18

**Authors:** A. Williams, M. Sittampalam, N. Barua, A. Mohd Nor

**Affiliations:** ^1^Department of Neurosurgery, Derriford Hospital, Plymouth, PL6 8DH, UK; ^2^Department of Neurology, Derriford Hospital, Plymouth, PL6 8DH, UK; ^3^Department of Neurosurgery, Frenchay Hospital, Bristol, BS16 1LE, UK

## Abstract

While ischaemic stroke remains a leading cause of death and disability, there have been recent advancements in treatment modalities including thrombolysis and decompressive hemicraniectomy. A retrospective review of patients treated in our NHS teaching hospital, in Plymouth (UK), over a 2 year period identified 17 thrombolysed patients, of whom two had undergone subsequent decompressive hemicraniectomy. These were non-dominant hemisphere strokes in young patients, aged 51 and 57. Initial NIHSS scores were 16 and 17, and they received thrombolysis at 2 hrs 42 min and 5 hrs 10 min post onset of symptoms respectively. CT imaging demonstrated cerebral swelling with significant midline shift in both cases, and decompressive hemicraniectomy was undertaken at 29 hrs 8 min and 27 hrs 30 min post-thrombolysis. We found no significant intra-operative complications attributable to prior use of thrombolytics. Both patients have had acceptable psychological and physical outcomes, with Barthel Index scores of 40 and 25, and MMSE scores of 29/30 and 27/30. We conclude that the use of thrombolytic therapy does not contra-indicate subsequent decompressive hemicraniectomy in well selected patients with non-dominant hemisphere strokes. More research in this field is required to elucidate factors which would facilitate recognition of stroke patients who will benefit most from aggressive medical and neurosurgical intervention.

## 1. Introduction

The acute treatment for ischaemic stroke, a leading cause of death and permanent disability in the Western world, underwent profound changes after the randomised double-blind NINDS study of intravenous thrombolysis in 1995 [1]. Despite its benefits, thrombolysis has remained an underused intervention perhaps due to its short window of efficacy, although this is being reconsidered [2], and delayed presentations to hospital are being addressed with multiple public health campaigns. Its benefits are, however, clearly documented. ECASS II [3], a multicentred European trial of 800 patients, using Alteplase (0.9 mg/kg) intravenously (IV), combined with strict CT and blood pressure inclusion criteria, supported the use of Alteplase within 3 hours of symptom onset. The potential benefits were counterbalanced by a 2- to 5-times increase in symptomatic intracranial haemorrhage. The NINDS study [1] suggested that intracerebral haemorrhage occurred in 5.8% of patients receiving tPA, which was associated with a 45% fatality rate.

In parallel with recent studies of thrombolysis, there has been an increase in interest in decompressive hemicraniectomy for anterior circulation ischaemic strokes since its initial description in 1935 [4]. Large cerebral infarcts are often associated with significant cerebral oedema, which may be significant enough to cause herniation and death. Hemicraniectomy was devised to counteract this postinfarction complication. This is most commonly a phenomenon of the second to fifth days after ictus [5], and half of deaths within the first month of young patients with ischaemic stroke can be attributed to space-occupying malignant infarction [6]. Again, this treatment is not without its controversies, the most predominant of which being that while it prevents death, it may promote life but with complete dependence. Recently, pooled data from three European multicentered, randomised, controlled trials (DECIMAL, DESTINY, and HAMLET) has suggested mortality and poor functional outcome can be improved by undertaking decompressive hemicraniectomy without increasing the numbers of completely dependent patients [7].

Combining these two interventions offers further opportunities to improve patient outcomes, but also raises further questions. Most predominant is the fear that precedent use of thrombolysis could predispose patients to excess risk of both intraoperative and postoperative haemorrhage. We sought to investigate this possibility in this retrospective analysis.

## 2. Methods

We examined clinical and surgical outcomes when decompressive hemicraniectomy was undertaken following intravenous thrombolysis in the same patients, and specifically when hemicraniectomy was undertaken for space-occupying cerebral oedema rather than post-thrombolysis haemorrhage. We analysed data from a cohort of patients treated in Derriford Hospital over a 30-month period from 13/05/2006 to 29/01/2009. Patients were identified by searching the hospital clinical coding database, using procedure codes for thrombolysis. The patients excluded either were thrombolysed for other reasons (e.g., myocardial infarction) or did not subsequently undergo hemicraniectomy. A total of 17 patients received intravenous thrombolysis between these dates, and two of these patients subsequently underwent decompressive hemicraniectomy. We undertook a detailed case notes study with interest focused on intraoperative and perioperative issues regarding the recent use of thrombolysis and patients' long-term outcome.

**Figure 1:**
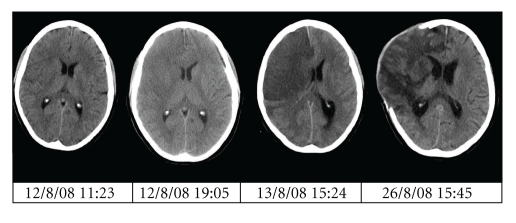


**Case 1 (SK (Figure 1))**

A 51-year-old right-handed female presented to the emergency department at 10:25 on 12/08/08, with a sudden onset left hemiplegia, occurring at 09:18, and involving the upper motor neurone of the VIIth cranial nerve and her upper and lower limbs, and a left-sided hemineglect. On admission her GCS was 15. Her past medical history included Hepatitis A in 1967. She had no medical allergies, and, aside from the oral contraceptive pill, she was on no medications. There was no contributory family history, and she was a life-long nonsmoker.

A noncontrast CT brain scan at 11:23 demonstrated characteristic grey and white matter changes in the right middle cerebral artery (MCA) territory and density within the right MCA, indicating acute infarct. There was no evidence of acute haemorrhage or hydrocephalus. Her admission blood profile was unremarkable (Hb 14.4, PLT 235, PT 12.8, APTT 23.2).

She was diagnosed with a right MCA ischaemic stroke, and, following appropriate consent, recombinant tissue plasminogen activator (rTPA), Alteplase was administered at 12:00. She was given a 5.4 mg bolus over 2 min, and then an infusion of 48.6 mg over the subsequent hour. A total of 54 mg rTPA was administered intravenously. Her blood pressure remained stable throughout.

Her neurological status remained unchanged until 13/08/08 when her GCS dropped to 9 (E3V1M5), and a CT brain scan (at 15:24) demonstrated an extensive right anterior cerebral artery (ACA) and MCA territory infarction. The associated mass effect resulted in 10 mm of midline shift to the left. There was radiological evidence of right uncal herniation. No intracranial haemorrhage was demonstrable.

After failed medical control of ICP, she underwent an emergency decompressive hemicraniectomy at 18:15 on 13/08/08. A standard operative protocol was used: a trauma flap was fashioned and frontotemporoparietal craniotomy was performed, and the dura was incised in a stellate pattern. The dura was left open, and an artificial dural patch (Dura-Guard, Synovis, St. Paul, USA) was laid over the defect. No subgaleal drain was sited. Routine bipolar diathermy was sufficient for haemostasis. This was an uncomplicated procedure. Her postoperative haemoglobin was 11.7 (preoperative was 14.4). No intraoperative blood component transfusions were administered, though, one day later, she received a 2-unit packed red cell transfusion.

Thereafter, she was managed on the intensive therapy unit (ITU) for medical optimisation of her intracranial pressure (ICP). Clexane 40 mg s/c, Aspirin 75 mg, and Clopidogrel 75 mg were commenced within a week. A routine postoperative scan on 26/08/08 was satisfactory. The evolution of radiological changes on CT scanning are shown in Figure 1.

She was transferred for physical and neurological rehabilitation three weeks after surgery. At her most recent outpatient review (6 months after surgery), her MMSE was 29/30. She was able to move from lying to sitting independently, to transfer from bed to chair and chair to car with the assistance of one, to stand with supervision of one, and to walk 20 metres with hand-rail assistance. She lives in a nursing home, with no difficulties with swallowing or meeting her nutritional needs, and she is continent. She had required antidepressant medication during rehabilitation, though this is no longer the case. She has a Barthel Index Score of 40.

**Figure 2:**
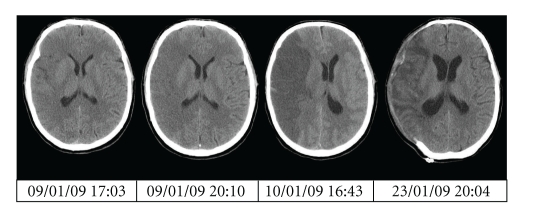


**Case 2 (DH (Figure 2))**

A 57-year-old right-handed male presented with sudden onset left hemiplegia at 13:30 on the 09/01/09 involving the upper motor neurone of the VIIth cranial nerve, and his left upper and lower limbs, but with a GCS of 15. His past medical history included right-sided sciatica and a childhood hypospadias repair. He had no medical allergies, nor used regular medications. He was a nonsmoker with an unremarkable family history.

A noncontrast CT brain scan at 17:03 demonstrated clear evidence of an acute, nonhaemorrhagic infarct in the right MCA territory, occupying greater than 50% of the usual MCA territory. Thrombus was seen in the right MCA. Routine investigations of blood parameters were unremarkable (Hb 14.9 PLT 242 PT 14.0 APTT 28.4).

He was consented for off-licence thrombolysis with Alteplase. This was commenced at 18:40 with a bolus of 6.3 mg over 2 minutes, followed by a 56.7 mg infusion over the subsequent hour. Thus, a total of 63 mg was prescribed intravenously. Blood pressure was monitored throughout the infusion, and there were no hypertensive episodes during administration.

The infusion was stopped early, after 53 mg total dose, as the patient developed a generalised tonic-clonic seizure. A repeat CT brain scan at 20:10 demonstrated evolving MCA territory malignant infarction with no evidence of acute haemorrhage, and the patient was subsequently intubated and ventilated on ITU.

Evolution of a fixed right mydriatic pupil was noted on the 10/01/09, and a repeat CT brain scan at 16:43 demonstrated that the cerebral oedema associated with the MCA infarct had significantly progressed with 12 mm shift in midline structures to the left. A left-sided intraparenchymal ICP bolt was inserted at 17:30, and ICP readings remained at 18–20 cm H20 despite optimal medical intervention.

On 10/01/09, a decision was made to proceed with decompressive hemicraniectomy due to refractory ICPs. A similar procedure was undertaken: the dura was opened, and an artificial dural patch (Dura-Guard) was laid over the defect. A subgaleal drain was sited for 48 hours. Bipolar diathermy was sufficient for haemostasis. There was a 220 mL blood loss intraoperatively. Postoperative Hb was 11.2 (14.3 preoperatively), and no intraoperative or postoperative blood component transfusions were required.

A routine postoperative scan demonstrated a maturing infarct involving the whole of the right MCA territory, but no intracranial haemorrhage. The shift of the midline structures had resolved, and the basal cisterns were patent. He was later discharged to intensive physical and neurological rehabilitation. Serial CT scan findings are shown in Figure 2.

On review nine months after stroke, his level of function allowed transfer from bed to chair with the assistance of one, and standing with two for short periods. He is independently mobile in a wheelchair. He is able to write independently, and his cognition is intact with only mild memory deficit (MMSE 27/30). There is no deficit in swallow safety, and no requirement for nutritional support. The cranial vault defect has been repaired with a titanium cranioplasty. He does, however, lack some insight into his physical condition and potential, and has required antidepressant medication. Due to impaired mobility, he has suffered with double incontinence. His Barthel Index Score is 25.

## 3. Discussion

Tissue plasminogen activator (t-PA) is an exogenous stimulator of the fibrinolytic system which degrades fibrin clots through activation of plasminogen to plasmin [8]. Plasmin not only lyses the fibrin component of a blood clot but also contributes to tissue remodelling by degrading extracellular matrix proteins, either directly or via the activation of matrix metalloproteinases [9]. 

In nonsurgically managed patients, serious complications of t-PA include systemic bleeding and anaphylaxis, but the most worrisome adverse reaction is intracerebral haemorrhage (ICH). Studies demonstrate symptomatic ICH occurs within 36 hours of treatment in 6.4% of patients treated with t-PA compared with 0.6% of patients treated with placebo. Despite this increased haemorrhage rate, mortality was similar in both groups with a trend toward increased mortality in the placebo group (21%) compared with the treatment group (17%) [1].

Moreover, there is a further concern that if neurosurgery is undertaken after intravenous thrombolysis, there may be an increased risk of perioperative haemorrhage, with significant risk of mortality. However, t-PA is rapidly cleared from the plasma at 550–680 mL/min, giving an initial distribution phase half-life of under 5 minutes, and a terminal elimination phase of approximately 40 minutes. Thus, 50% of t-PA is cleared from the plasma within 5 minutes after discontinuation of an IV infusion, and approximately 80% is cleared within 10 minutes. 

Malignant infarction and resultant space-occupying cerebral oedema is most common between the second and fifth days postictus [5], and, given the short serum half-life of t-PA, it is likely that any excess haemorrhage risk has returned to baseline at the time when neurosurgical intervention is being considered. However, there is little experimental or anecdotal evidence to confirm this hypothesis at present, and the evidence will need to be examined in greater detail before definitive conclusions on the safety of major neurosurgical intervention following thrombolysis can be reached. 

This study has identified two cases in which both thrombolysis and decompressive hemicraniectomy have been undertaken in the same patients. In both cases, the patients rapidly underwent thrombolysis, and subsequently underwent emergency neurosurgery, and both patients survived with the avoidance of a vegetative state (Table 1). Furthermore, neither patient suffered any haemorrhagic complication of undergoing both interventions. 

A recently published study [10] of mortality following stroke reported 17 patients who underwent hemicraniectomy following initial thrombolysis. It was demonstrated that thrombolysis prior to hemicraniectomy had no effect on inpatient hospital mortality (odds ratio 1.21, 95% confidence interval 0.43, 3.39). In support of this finding, our case reports may point the way for further studies into the role of neurosurgical intervention following intravenous thrombolysis. While it is impossible to make any definitive conclusions from a case series of two patients, we found no evidence that hemicraniectomy is associated with excess haemorrhage risk in patients receiving prior thrombolysis. More research into these treatments is needed to further elucidate the potential for both modalities to be used in appropriate patients.

Despite these successful cases, our findings clearly have caveats. These are both young patients, and selection for such aggressive interventions is appropriately biased in favour of the younger patient in whom the outcomes after stroke are demonstrably better. Both were nondominant hemisphere stokes, and clearly remained significantly disabled. Therefore, it would be unwise to extrapolate any findings to dominant hemisphere strokes.

## 4. Conclusions

Intracerebral haemorrhage is a potentially fatal complication of intravenous thrombolysis. However, there is very little evidence to support or refute the hypothesis that prior thrombolysis results in excess haemorrhage risk for patients who suffer space-occupying cerebral oedema and are selected for subsequent decompressive hemicraniectomy. Decompressive hemicraniectomy following thrombolysis represents an aggressive treatment paradigm, and the decision to proceed with such intervention requires a multidisciplinary team approach and a frank and open discussion with patients and/or their relatives. Whilst decompressive hemicraniectomy may preserve both life and function in well-selected patients, the potential risks of surgery, including life-threatening haemorrhage, must be discussed with patients and/or relatives in order to obtain a fully informed consent. This case series supports the hypothesis that decompressive hemicraniectomy following intravenous thrombolysis is not associated with excess haemorrhage risk. However, definitive guidelines must be based on larger prospective clinical studies.

## Figures and Tables

**Table 1:** 

	Patient 1	Patient 2
Age	51	57
Time from onset of symptoms to arrival to hospital	1 hr 7 min	2 hrs 8 min
NIHSS scores on admission	16	17
Time from onset of symptoms to thrombolysis	2 hrs 42 min	5 hrs 10 min
Time from onset of symptoms to operation	32 hrs 57 min	32 hrs 40 min
Time between thrombolysis and operation	29 hrs 8 min	27 hrs 30 min
Barthel index scores	40	25
Mini-mental state examination	29/30	27/30
